# An Intelligent Approach for Cloud-Fog-Edge Computing SDN-VANETs Based on Fuzzy Logic: Effect of Different Parameters on Coordination and Management of Resources

**DOI:** 10.3390/s22030878

**Published:** 2022-01-24

**Authors:** Ermioni Qafzezi, Kevin Bylykbashi, Phudit Ampririt, Makoto Ikeda, Keita Matsuo, Leonard Barolli

**Affiliations:** 1 Graduate School of Engineering, Fukuoka Institute of Technology (FIT), 3-30-1 Wajiro-Higashi, Higashi-Ku, Fukuoka 811-0295, Japan; mgm19201@bene.fit.ac.jp; 2 Department of Information and Communication Engineering, Fukuoka Institute of Technology (FIT), 3-30-1 Wajiro-Higashi, Higashi-Ku, Fukuoka 811-0295, Japan; kevin@bene.fit.ac.jp (K.B.); makoto.ikd@acm.org (M.I.); kt-matsuo@fit.ac.jp (K.M.); barolli@fit.ac.jp (L.B.)

**Keywords:** VANETs, IoV, SDN, fuzzy logic, cloud computing, fog computing, edge computing, resource management, predicted contact duration

## Abstract

The integration of cloud-fog-edge computing in Software-Defined Vehicular Ad hoc Networks (SDN-VANETs) brings a new paradigm that provides the needed resources for supporting a myriad of emerging applications. While an abundance of resources may offer many benefits, it also causes management problems. In this work, we propose an intelligent approach to flexibly and efficiently manage resources in these networks. The proposed approach makes use of an integrated fuzzy logic system that determines the most appropriate resources that vehicles should use when set under various circumstances. These circumstances cover the quality of the network created between the vehicles, its size and longevity, the number of available resources, and the requirements of applications. We evaluated the proposed approach by computer simulations. The results demonstrate the feasibility of the proposed approach in coordinating and managing the available SDN-VANETs resources.

## 1. Introduction

According to World Health Organization, around 1.3 million people die every year because of road traffic crashes [1]. The key risk factors come from human error (speeding, wrong decisions, etc.), irresponsible behavior (drinking, distracted driving, fatigue, etc.), unsafe road infrastructure, and bad weather conditions (e.g., inadequate visibility and slippery roads) [2,3,4]. Vehicular Ad hoc Networks (VANETs) have emerged as a solution to alleviate all these factors by means of different applications [5,6,7]. For example, implementing an accident prevention system in VANETs that considers velocity, weather condition, risk location, nearby vehicles density, and driver fatigue can reduce the number of road crashes and consequently the number of deaths [8]. Other applications, on the other hand, can improve traffic management and the driving experience [9,10,11].

Nevertheless, safety and traffic management are correlated to each other in many ways. For instance, traffic congestion leads to a higher risk for car crashes, as drivers are prone to drive faster in order to compensate for the delay caused by traffic [12]. There exist many vehicular navigation systems, with Google Maps being the most popular, which recommend alternative routes to vehicles to avoid traffic congestion. Such navigation systems compute different alternative routes in terms of shortest driving distance, shortest driving time and lowest driving cost (in case of toll roads) [13]. However, taking into account merely these parameters does not actually achieve the goals of traffic management systems as navigation systems only serve each vehicle individually, thus offering better services only for their drivers. VANETs, on the other hand, accomplish substantially bigger goals. They benefit all road users, without any exception, mostly by depending only on content awareness and inter-vehicle communications. Context-awareness is expected to play a key role in VANETs, not only by ameliorating traffic management, but also other important metrics such as the driving experience, the safety of road users, and the environmental impact [14]. The inter-vehicle communication in VANETs enables a broader horizon of awareness for the state of other vehicles in the network and the condition of the surrounding environment. Such data include information about traffic lights, weather conditions, public safety information, and so on. Furthermore, the response time in VANETs is dramatically reduced as vehicles would be notified in real time, almost immediately after some situation occurs [15,16,17].

In terms of networking, VANETs can be defined as networks of vehicles spontaneously created, able to connect vehicles with other vehicles in the network and with the infrastructure via Vehicle-to-Vehicle (V2V) and Vehicle-to-Infrastructure (V2I) communication links [18]. They are a subclass of Mobile Ad hoc Networks (MANETs), and as such are based on inter-vehicle communication and do not rely on central coordination [19]. The vehicles behave as sensor nodes and relay the messages via one-hop or multi-hop communications. The infrastructure includes Road Side Units (RSUs), road signs, Electronic Toll Collection (ETC), and so on. For example, RSUs are deployed along the roads and are used to increase robustness, connectivity, and coverage by acting as static sensors and relay nodes [20].

The evolution of the 5th Generation of Cellular Networks (5G) marks a huge leap in the advance of VANETs. 5G base stations (5G-NR gNodeB) can serve as a gateway to the Internet and therefore enable big data storage, processing, and analyzing in the cloud infrastructure [20]. Due to the integration of Artificial Intelligence (AI), cloud platforms will be able to take better decisions to enhance the driving experience. Vehicles will be able to exchange information with many more entities, such as pedestrians, infrastructure, and networks, via Vehicle-to-Everything (V2X) communications [21]. With all these entities connected through vehicles and with many others being designed for the Internet of Things (IoT), the term ad hoc was considered obsolete by many researchers as it does not comprehensively cover the wide range of technologies involved within/connecting these entities [22,23,24,25]. The concept of the Internet of Vehicles (IoV) has emerged as a broader concept to better represent the new era of vehicular networks [26]. However, since IoV is somewhat equivalent with the novel VANET architectures, which are very different from traditional VANETs, in this work we consider these two concepts interchangeable.

Even though the IoV will open many possibilities for a plethora of applications, there are still many challenges that are yet to be addressed [7,10,27,28]. Of the utmost importance is, for example, the management of the abundant information and resources available in these networks. Even a single vehicle generates huge amounts of data and considering the fact that the number of vehicles keeps increasing, managing these networks becomes more difficult. In addition, many new applications that require more and more resources come along continually, leading to increased complexity in network management.

In this work, we deal with the resource management problem for which we propose an intelligent architecture based on Fuzzy Logic (FL) and Software Defined Networking (SDN) approaches that can efficiently manage cloud-fog-edge storage, computing, and networking resources in VANETs. By using FL, the proposed approach can manage the resources in real-time while dealing with imprecision and uncertainty. The contributions of the work are summarized as follows:

- The paper presents an integrated system, called Integrated Fuzzy-based System for Coordination and Management of Resources (IFS-CMR), which, different from existing approaches, makes a decision following a bottom-up approach in a cloud-fog-edge architecture.
- IFS-CMR considers the condition of the network created between vehicles, such as the Quality of Service (QoS) in the network and the unused amount of resources, together with the application requirements, to select the best resources for a particular situation.
- IFS-CMR is composed of three subsystems, namely Fuzzy-based System for Assessment of QoS (FS-AQoS), Fuzzy-based System for Assessment of Neighbor Vehicle Processing Capability (FS-ANVPC), and Fuzzy-based System for Cloud-Fog-Edge Layer Selection (FS-CFELS), each having a key role in the proposed approach.
- The feasibility of the proposed architecture in coordinating and managing the available VANETs resources is demonstrated by the results of extensive simulations.

The rest of this paper is organized as follows. Section 2 provides a background overview of the emerging technologies integrated within VANETs which enable the full implementation of our proposed system, as well as a short review of several research papers relevant to this work. The proposed approach and the details of its implementation are presented in Section 3. Section 4 discusses the evaluation results. The last section, Section 5, gives some concluding remarks and ideas for future work.

## 2. Background Overview

In this section, we discuss different technologies, paradigms, and approaches that enable emerging applications of vehicular networks. We give an overview of the advantages that IoT, cloud-fog-edge computing, and SDN bring in VANETs, together with some of their challenges and solutions. Lastly, we review several related research works.

### 2.1. Internet of Things

IoT is the network of everyday objects that connect over the Internet with other devices or with other networks, for the purpose of monitoring and controlling these objects, among others. The IoT devices are embedded with sensors and a communication unit, which enable gathering and sharing information with each other in order to achieve a common goal. Vehicles can also connect to the Internet through cellular wireless technologies and interact with other networks of IoT. The integration of connected vehicles with IoT brings many advantages, as vehicular networks can use the information made available from other integrated components. For example, drivers will get real-time information about traffic, weather conditions, or the condition of a remote road. Therefore, better safety and traffic management can be achieved even in terrains where inter-vehicle communication is impossible or the IoV infrastructure is lacking. On the other hand, other networks can exploit the information coming from vehicular networks, too. Despite the attractive features, IoT faces scalability problems as the number of connected IoT devices increases dynamically.

### 2.2. Cloud, Fog, and Edge Computing

Cloud computing offers storage and computation facilities that are placed remotely, at an extended distance from vehicles, typically in cloud data centers. They offer unlimited storage and computational capability that can be accessed from anywhere. Therefore, vehicles can send and retrieve huge amounts of data at any time and place, without being concerned about their limited storage capability. However, when it comes to time-sensitive applications, the distant cloud is not able to fulfill the latency requirements.

Due to the dynamic nature of vehicles, it is necessary to deliver the applications with minimal delay and ensure uninterrupted service. To address these issues, a computing paradigm that takes place closer to the vehicles is needed. Fog computing is physically located somewhere between the edge layer and cloud layer and bridges the gap between the two. It ensures abundant storage, computational and network resources, real-time communication, high bandwidth, high mobility support, and context awareness [29,30,31]. Moreover, because of the proximity to the vehicles, fog computing is a good solution for services that require high QoS.

On the other side, smart vehicles have a considerable amount of storage and computing capabilities, and therefore can be considered a form of edge computing. While some resources are reserved for the operating system, other available resources can be used for delivering VANET applications and processing data obtained from sensors. Processing and analyzing data at the edge will avoid the massive traffic flow in the core network, which occupies much of the already limited network resources.

### 2.3. Software Defined Networking

SDN is recently adopted in VANETs to deal with network management issues because it is an approach that has a better view of the network, and therefore offers higher flexibility, more scalability, and better programmability. SDN is a mechanism that takes advantage of the decoupling between the control plane and data plane, allowing for complex network management. For such dynamic networks like VANETs, with an abundance of vehicles that cause frequent topology changes and the presence of heterogeneous nodes, SDN can make better decisions for coordination and management of resources, since it considers the requirements of the entire network and avoids interference with other networks. In addition, greater control is achieved by comprehensively knowing the requirements of the VANET applications and by exploiting the available resources of the network [32]. The network adapts to the dynamic changes of the network and also supports the emergency situations by prioritizing the application requirements in terms of bandwidth, propagation, delay, and processing resources.

### 2.4. Vehicular Ad Hoc Networks

Given that vehicular networks are a subset of wireless networks, some challenges are common to other existing wireless networks, including limited transmission ranges, the limited number of communication channels, and interference. However, there are many other challenges that come from the unique characteristics of vehicular environments. Large and dynamic topologies, variable capacity wireless links, bandwidth and hard delay constraints, and short contact durations are some of the characteristics of these networks. These challenges are caused by the high mobility and high speed of vehicles, and frequent changes in density happening even in the same area. For example, the vehicle density is higher on main streets than on secondary ones and it changes sharply over time (the same streets are busier during peak hours as opposed to night hours or other parts of the day).

**Figure 1:**
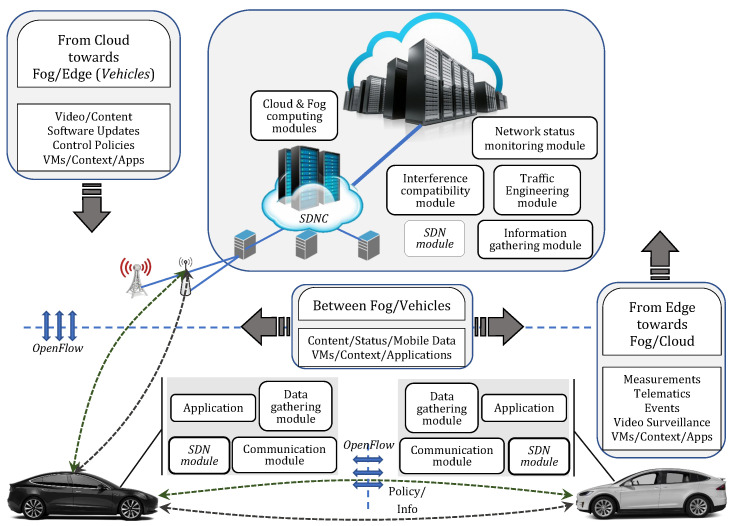
Infrastructure and content distribution in the cloud-fog-edge SDN-VANETs.

In addition, as the number of smart vehicles and sensors incorporated in vehicles keeps increasing, a huge amount of data will be generated in VANETs. Conventional VANETs, which are based on vehicle-to-vehicle communication and depend only on self-contained resource capabilities, lack the needed resources for dealing with such massive amounts of data. Moreover, the lack of a centralized management entity makes it hard for such dynamic networks to have an equitable share of resources and therefore results in collision of transmitted packets and less efficient use of channel resources [20]. With the integration of cloud-fog-edge computing and the SDN approach within VANETs, prospective solutions in managing the aforementioned problems can be achieved. The main components of this architecture together with the content flow are illustrated in Figure 1.

The vehicles are equipped with limited computing and storage resources and after collecting information from the data gathering module, the vehicles can perform real-time data analysis for non-complex data. In this way, the information is processed locally and there is no delay in case immediate action is needed. However, the limitations of edge computing make it necessary for VANETs to rely upon fog and cloud computing. Fog computing in VANETs is the extension of the cloud paradigm brought in proximity of the vehicles, which offers predictable latency and seamless resource management by supporting mobility and geo-distribution [33].

The computation of complex data, which are beyond the capability of edge and fog layer and are not time-sensitive, can be sent to the cloud layer for further advanced analysis that takes a long time and requires abundant storage. They are also used as a repository for control policies, software updates, and so on. The integration of SDN in VANETs handles the issues of such large-scale, heterogeneous, and dynamic networks by providing a robust mechanism for data traffic control and resource management of all the components of this novel architecture of VANETs. The implementation of cloud-fog-edge and SDN in VANETs will enhance VANETs services and will pave the way for future applications.

### 2.5. Related Works

Several researchers have considered bringing computing and storage capabilities closer to the edge of vehicular networks. In [34], the authors emphasize the importance of Multi-Access Edge Computing (MEC) as a crucial paradigm in IoV for supporting heterogeneous devices and emerging 5G services. In [35], the authors discuss how to provide vehicular networks with cloud computing services and introduce a new paradigm of the virtual cloud computing architecture based on a Macro-Micro-Cloud novel approach that allows hierarchically integrating available resources from vehicles into the edge computing system, with the aim of managing data, reducing communication complexity, and improving QoS.

A resource management scheme based on FL is proposed by Miao et al. [36] to manage resources (text, audio, and video) in VANETs, under a fog computing platform. The fuzzy logic system determines the survival time of the resources of a local server based on a data set of request time and download time for each resource, which is recorded by a V2I communication model. In this way, the system keeps updated information about the availability of resources in real-time. The simulation results show that the proposed model manages the resources effectively and satisfies the needs of clients with resource sharing in the case of dynamic topology, intermittent connectivity, or limited storage capability of local servers. In [37], the authors deal with the problem of maintaining the QoS during service migration from one node to another by targeting resource management strategies at fog nodes. The authors introduce two schemes that prioritize selected services. The first scheme reserves fog resources based on traffic load, while the second scheme frees fog resources allocated for low-priority and reallocates them for high-priority services. Both schemes show an increase in one-hop access probability, especially for high-priority services. Khan et al. [38] give another perspective for resource allocation strategies in VANETs. The authors propose a Hybrid-Fuzzy Logic guided Genetic Algorithm (H-FLGA) approach for the SDN controller, to solve a multi-objective resource optimization problem for 5G driven VANETs. The hybrid system is based on the service requirements of customers and good results are achieved in terms of capacity, delay, traffic load, energy optimization, cost, and so on.

Mendiboure et al. [39] analyze the main features of edge computing and classify the appropriate technology for different vehicular applications by comparing the network capacities with the applications’ requirements. To fully support the various applications and services, the network architectures must satisfy specific QoS requirements while exploiting the available resources. In [40], the authors introduce a fuzzy-based system to estimate QoS for different broadcasting protocols in VANETs. The broadcasting protocols are suitable for delivering infotainment and road condition information to all vehicles in the network without exception. However, they cause problems such as broadcast storms. The authors compare the results of several QoS parameters such as delay, reachability, packet delivery ratio, and overhead value. Even though these are important parameters to estimate the network performance, different VANET applications have different requirements, which means the network performance changes in regard to the application requirements.

In previous works, we have considered the resource management problem in vehicular networks and have proposed different intelligent approaches to the problem. In [41], we have proposed a simple fuzzy-based system that provides vehicles with a recommendation on the storage resources they can use based on factors such as data size and time sensitivity. The storage resources consist of those of the nearest vehicle, fog server, and cloud data center. In [42], we improved our cloud-fog-edge layered architecture by leveraging the SDN and the global overview of the network that this approach enables; thus, providing the system with more resources, without restricting it to only one adjacent vehicle or fog server. The effect of the number of neighboring vehicles comprising the edge layer was observed in [43]. The results showed that the system tends to choose the edge layer when more vehicles are nearby. Different from these works, in this paper, we present a more complete approach to the resource management problem, by taking into consideration more parameters that better represent the characteristics of each layer and the application requirements.

## 3. Proposed Architecture

**Figure 2:**
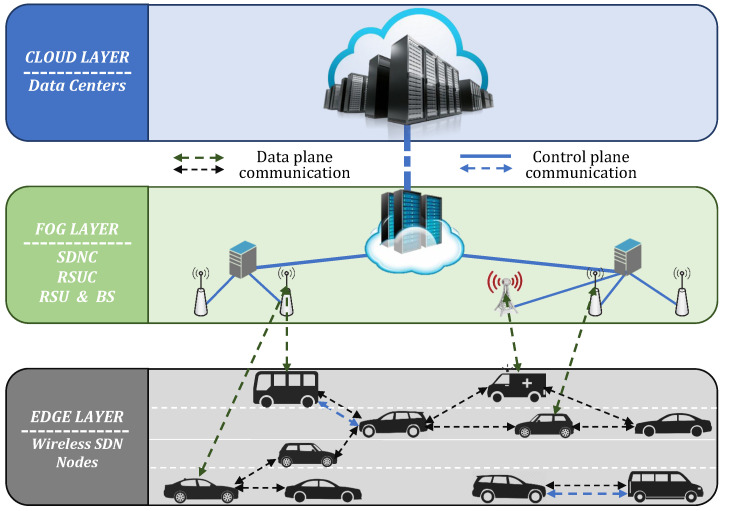
Layered architecture of cloud-fog-edge SDN-VANETs.

This section presents the architecture of our proposed approach for coordination and management of VANETs resources. The information acquired by many vehicles, not only by an individual vehicle, increases the accuracy; thus, better decisions and predictions can be made. However, there is a major problem in gathering, processing, and analyzing the enormous amount of data generated while keeping the network cost at a minimum. Managing the resources of the network while providing the application’s requirements is yet another challenge. Our proposed approach addresses these issues. The proposed approach considers a layered cloud-fog-edge SDN architecture that is coordinated by a fuzzy system implemented in the SDN Controller (SDNC) and SDN modules. This architecture is illustrated in Figure 2.

SDNC manages the resources of the edge, fog, and cloud layer and determines the appropriate layer for data storage and computing, based on the output of the fuzzy system. The edge layer includes the resources of all On-Board Units (OBUs) of the smart vehicles that are able to communicate with each other. The vehicles act not only as a relay node, but they also process and analyze the data by themselves and at the same time share their available resources with some vehicle that has a shortage of resources (hereinafter will be referred to as *the vehicle*). The fog layer consists of the RSUs, RSU Controllers (RSUCs), Base Stations (BSs), and fog servers, which are ideally only a few hops away from the vehicles. It offers more resource capabilities, compared to the edge layer, while still providing computing in real-time. Whereas the cloud layer offers abundant storage and computing facilities, located far away at the cloud data centers.

We consider this architecture from a bottom-up approach, which implies that the edge layer is the first layer considered, based on the available connections and service requirements. If the application requirements are not fulfilled or there are no or only very few available connections, then the fog layer is the layer taken into consideration. For instance, safety applications are suitable for either edge layer or fog layer, as both these layers support real-time processing and high QoS. The cloud layer is used to process applications that are delay tolerant and require long-term analytics. Through this approach, all network resources are utilized effectively and massive traffic flow in the core network is avoided.

IFS-CMR is implemented in the SDNC and the vehicles which are equipped with an SDN module. In case a vehicle does not have an SDN module, it sends the information to SDNC which computes the needed information and sends back its decision.

In the next subsections, we give details of the composition of the proposed approach and describe the input and output parameters of each subsystem. In addition, we present the data gathering and communication module which is integrated into the vehicles and explain the FL Controllers (FLCs) of the system.

### 3.1. Data Gathering and Communication Module

**Figure 3:**
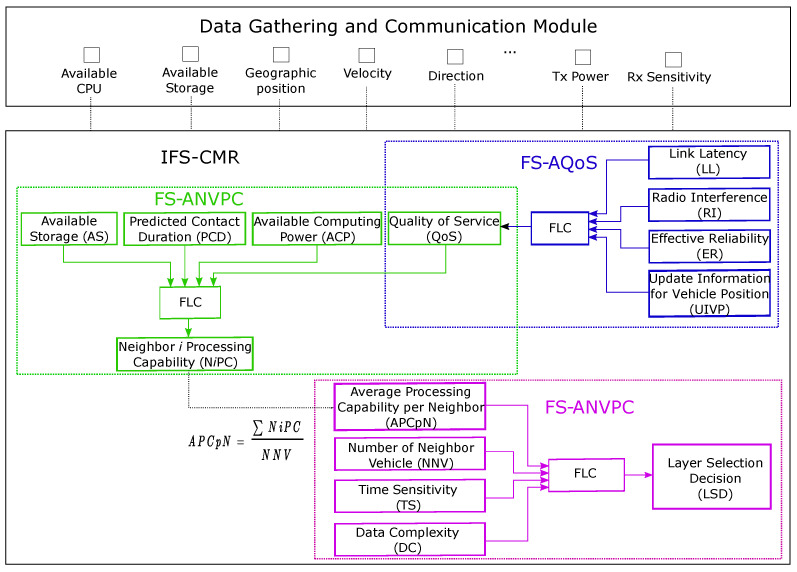
Structure of fuzzy integrated system.

The vehicles are equipped with various sensors (lidar, radar, ultrasonic, camera, etc.) which are placed internally and externally to acquire information about the vehicle itself (its speed, direction, steering wheel movements, tire slip, distance between the lane and other nearby vehicles, and so forth) and the condition of roads (congested roads, inadequate traffic signs, potholes, ice patches, or other hazards); a wireless transceiver device which supports different wireless technologies that enable communications with other entities; a GPS device that provides precise information about location; and an OBU which controls the communication of vehicle with other entities and offers computing, storage, and networking facilities [44]. We have included all these components in the data gathering and communication module, as shown in Figure 3 (also referred to separately as data gathering module and communication module in Figure 1).

Once the data is sensed and processed, the vehicles then share with one another important information through beacon messages. They broadcast these beacons periodically to other vehicles via V2V communication links to increase cooperative awareness between them. The beacon messages are critical for our approach as well. Once the vehicles receive them, they extract the information they need, which for IFS-CMR is about their neighbors’ geographic position, speed, direction, transmission power, available storage, available computing power, etc. Then, IFS-CMR makes the necessary calculations to update the current condition of all the input parameters.

### 3.2. IFS-CMR Parameters

The parameters of IFS-CMR are described in detail in the following.

Link Latency (LL): Latency is a strong requirement in VANETs. Many applications, especially those related to safety, must run in real-time and the network must provide a very low latency despite the rapid and high topology changes. Therefore, the time it takes the first bit to get from the sender to the destination is crucial in providing high QoS.

Radio Interference (RI): Radio Interference indicates the unwanted signals that come from transmissions of adjacent vehicles and disrupt the reception of information. These signals can cause problems from low data speed transmissions to even complete loss of information.

Effective Reliability (ER): We define effective reliability as the capacity of the network to successfully deliver messages to its destination. There are many factors that influence ER that include the bandwidth of transmission medium, number of collisions, and buffer size, among others.

Update Information for Vehicle Position (UIVP): It is necessary for vehicles to have the coordinates of other surrounding vehicles in order to detect dangerous situations or to monitor traffic. However, too many packets occupy more bandwidth, whereas few packets cannot accurately discover the position of neighboring vehicles.

Quality of Service (QoS): Each application has different requirements in terms of latency, bandwidth, throughput, and so on. Safety applications, for example, require real-time communications over reliable links. The latency constraints for such applications are in the range of a few milliseconds [45,46]. However, satisfying QoS requirements at all times is not a straightforward task given the highly dynamic topology and interference on these networks.

Available Computing Power (ACP): Recent VANET applications require significant computational resources and real-time processing. The intelligent vehicles in the new generation of VANETs are capable of handling some of these applications. Vehicles use their computing power to run their own applications, but they can also allocate some of it for other vehicles to help them in case they need additional computing power to complete certain tasks. When vehicles are willing to share their resources, they let their neighbors know by sending them information about the amount they want to share. In other words, they decide the number of physical processor cores and the amount of memory that other vehicles can use.

**Figure 4:**
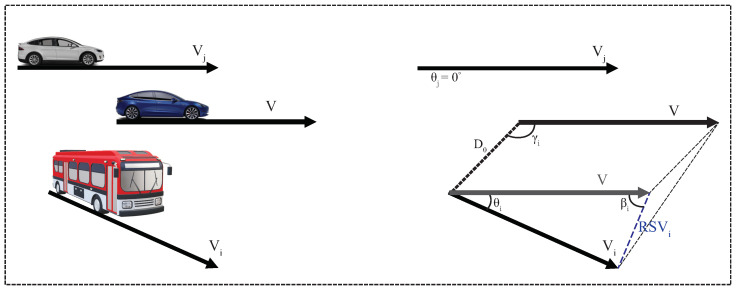
Graphical representation of vehicles moving at different velocities and directions.

Predicted Contact Duration (PCD): In a V2V communication, the duration of the communication session is important since it determines the amount of data to be exchanged and the services that can be performed. A vehicle in need of additional resources (*the vehicle*) will have to set up virtual machines on the neighbors that are willing to lend their resources; therefore, the contact duration becomes even more important since much more time is needed to accomplish these tasks than just performing a data exchange. Since the vehicles change their direction or speed, we can only make a prediction of their contact duration based on the value of the parameters at the time when the beacon message was transmitted (for more accuracy, the PCD is updated each time a new beacon message from that neighbor is received). To calculate the PCD between *the vehicle* and a neighbor vehicle *i* (see Figure 4 for illustration), we first calculate the relative speed between these two vehicles using the law of cosines, as given in Equation (1).

(1) $$RSV_{i}=\sqrt{V^{2}+V_{i}^{2}-2VV_{i}\cos\theta_{i}}$$

where *V* is the speed of *the vehicle*, $V_{i}$ is the speed of neighbor *i*, and $\theta_{i}$ is the angle between their directions. Then, we use the law of cosines once again to calculate the PCD, as given in Equation (2).

(2) $${\left(RSV_{i}\cdot PCD\right)}^{2}+D_{0}^{2}-2\left|RSV_{i}\right|\cdot PCD\cdot D_{0}\cos\left(\gamma_{i}+\beta_{i}\right)=CR^{2}$$

where $D_{0}$ denotes the initial distance between the two vehicles, *CR* is the communication range, $\gamma_{i}$ is the angle between the direction of *the vehicle* and $D_{0}$ imaginary line; whereas $\beta_{i}$ is calculated with the Equation (3), which is derived from the law of sines.

(3) $$\beta_{i}=\left\{\begin{array}{cc} \arcsin(\frac{V_{i}\sin\theta_{i}}{|RSV_{i}|}), & \mathrm{for}\;V_{i}\leq \sqrt{V^{2}+RSV_{i}^{2}}\\ 180{}^{\circ}-\arcsin(\frac{V_{i}\sin\theta_{i}}{|RSV_{i}|}), & \mathrm{for}\;V_{i}>\sqrt{V^{2}+RSV_{i}^{2}},\theta \geq 0\\ -180{}^{\circ}-\arcsin(\frac{V_{i}\sin\theta_{i}}{|RSV_{i}|}), & \mathrm{for}\;V_{i}>\sqrt{V^{2}+RSV_{i}^{2}},\theta <0 \end{array}\right.$$

We posit that when two vehicles are getting farther from each other from different directions, their directions form a positive angle, whereas when the vehicles are getting closer, θ is negative.

Available Storage (AS): Since vehicles generate and receive enormous amounts of data, their storage might be insufficient despite their large storage resources. If *the vehicle* needs to use also the storage of the neighbors, that should be large enough to allow *the vehicle* to run the virtual machine. This storage is used also to store data after completing specific tasks of all the tasks these neighbors are asked to accomplish.

Neighbor i Processing Capability (NiPC): Describes the capability of a vehicle to help another *vehicle* that lacks the appropriate resources to accomplish certain tasks. The values of this parameter range between 0 and 1, with the value 0 implying that the neighbor cannot help at all and 1 that the neighbor is in the best condition to help out *the vehicle*.

Average Processing Capability per Neighbor Vehicle (APCpNV): This parameter is the average of the Processing Capability (PC) of all neighboring vehicles within *the vehicle’s* communication range. It is an important parameter that represents the capability of the edge layer, and it is calculated as the sum of the PC of each neighbor vehicle divided by the number of neighboring vehicles.

Number of Neighboring Vehicles (NNV): This parameter changes continuously due to the vehicles moving out of *the vehicle’s* communication range and the ones that appear. Vehicles traveling at the opposite direction lead to even more frequent changes. Since the bigger the angle between the directions, the bigger the distance created between the vehicles, we consider only the neighbors whose directions with the direction of *the vehicle* create angles that are smaller than 90°. Vehicles traveling in directions that create bigger angles move out of the communication range very quickly, making it impossible for *the vehicle* to use their resources.

Time Sensitivity (TS): Different applications have different requirements in terms of latency. For instance, safety applications require a strict latency to be guaranteed, ideally <1 ms, whereas comfort and entertainment applications can tolerate latencies up to some seconds and are considered delay-tolerant [45,46]. System updates and the data collected for long-term analytics can tolerate even longer latencies, thus for such applications, the latency is not considered a requirement at all.

Data Complexity (DC): There are many factors that dictate the data complexity, and the volume is only one of them. Even a single application might use data that differ in type and structure, not to mention that they may come from many disparate sources (e.g., sensors, cameras, radar, lidar). Besides, there are different kinds of applications that include not only VANET applications, as in the Big Data era vehicles can also be used to compute data non-related to VANETs. However, not all the data need considerable processing as some of them are in the form of small messages that are used to inform the vehicles for particular situations.

Layer Selection Decision (LSD): The output parameter values, which are always between 0 and 1, denote three decisions—the interval [0, 0.3] indicates that *the vehicle* can use the edge layer resources, the values in the interval (0.3, 0.7) specify that the layer to be used is the fog layer, whereas the values in [0.7, 1] specify the cloud layer as the most appropriate layer to run their applications.

### 3.3. Description of IFS-CMR Subsystems

The input parameters of each subsystem of IFS-CMR do not correlate to one another, leading to an NP-hard problem. Problems with three or more uncorrelated parameters are classified as NP-hard because finding a mathematical model that can calculate an output in polynomial-time is practically impossible. Heuristic or meta-heuristic approaches are proven to provide adequate solutions for these kinds of problems, but each method has a limited scope to which it can be applied. For instance, genetic algorithms give good solutions for optimization and allocation problems. Neural networks can be applied to recognition problems and rule learning. FL, on the other hand, can be used to provide a solution for decision-making and control problems in real-time, especially when the system contends with high levels of imprecision and uncertainty [47,48,49,50,51,52].

Making real-time decisions while dealing with imprecision and uncertainty is the advantage of IFS-CMR. The characteristics of the network created between vehicles change continually and rapidly; therefore, the resources must be managed in real-time. Moreover, because of the rapid changes, the decision is reached in presence of much imprecision.

IFS-CMR is comprised of three integrated subsystems (FS-AQoS, FS-ANVPC, and FS-CFELS), each controlled by its respective FLC. Each subsystem has a key role in the system. They have their own input parameters and their output serves the subsystem that follows. The structure of IFS-CMR is shown in Figure 3. The way we have built IFS-CMR has allowed us to continually improve it by discovering the implementation flaws (contrary to many AI systems, which behave as black boxes that do not provide any feedback how they reach their decision, and consequently are difficult to improve).

**Figure 5:**
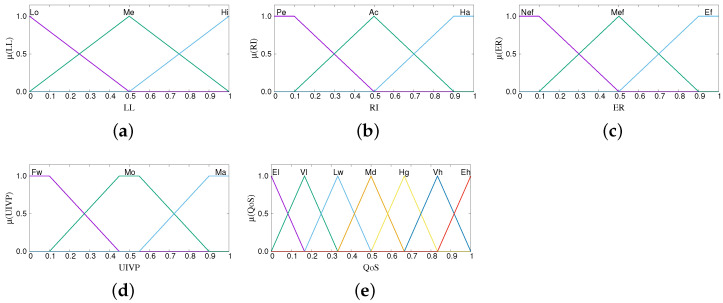
Membership functions of FS-AQoS. (**a**) Link Latency, (**b**) Radio Interference, (**c**) Effective Reliability, (**d**) Update Info. for Vehicle Position, and (**e**) Quality of Service.

**Figure 6:**
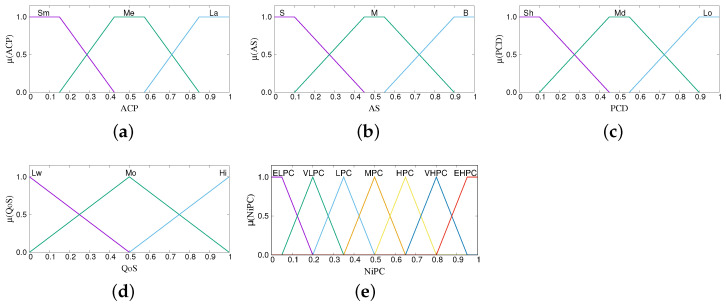
Membership functions of FS-ANVPC. (**a**) Available Computing Power, (**b**) Available Storage, (**c**) Predicted Contact Duration, (**d**) Quality of Service, and (**e**) Neighbor *i* Processing Capability.

**Figure 7:**
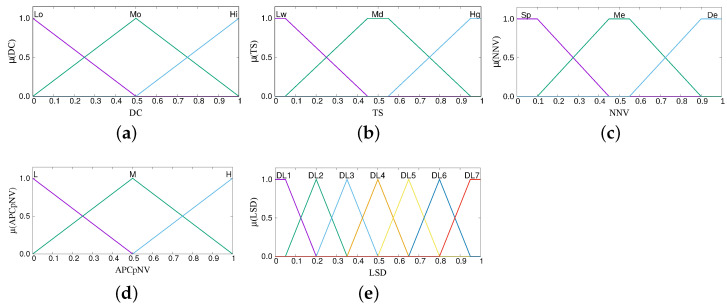
Membership functions of FS-CFELS. (**a**) Data Complexity, (**b**) Time Sensitivity, (**c**) Number of Neighboring Vehicles, (**d**) Avg. PC per Neighbor Vehicle, and (**e**) Layer Selection Decision.

The term sets for the parameters of each subsystem are shown in Table 1, Table 2 and Table 3. The parameters are fuzzified using the membership functions shown in Figure 5, Figure 6 and Figure 7. The number of terms for each parameter and the characteristics of each membership function are determined through the experience gained by running many simulations. From our experience, using less than three linguistic terms for an input parameter has the risk of inefficient control and making poor decisions, whereas using more leads to redundancies and increased complexity. The same holds true for the overlap of membership functions. Less overlap results in poor decisions, more overlap brings redundancies. Regarding the shape, we use triangular and trapezoidal membership functions as they are the most suitable ones for real-time operation.

In Table 4, Table 5 and Table 6, we show the Fuzzy Rule Base (FRB) of FS-AQoS, FS-ANVPC, and FS-CFELS, respectively. The FRB forms a fuzzy set of dimensions $|T\left(x_{1}\right)|\;\times \;|T\left(x_{2}\right)|\;\times \;\cdots \;\times \;|T\left(x_{n}\right)|$, where $|T(x_{i})|$ is the number of terms on $T(x_{i})$ and *n* is the number of input parameters. Therefore, since each subsystem has four input parameters with three linguistic terms, each FRB consists of 81 rules. The control rules have the form: IF “conditions” THEN “control action”. For instance, for FS-AQoS, for Rule 20: “IF LL is Lo, RI is Ha, ER is Nef and UIVP is Mo, THEN QoS is Md”.

## 4. Simulation Results

In this section, we discuss the simulation results of IFS-CMR. The results for each subsystem are separately organized to better present and understand the way IFS-CMR controls its final output, which is the selection of the resources to be used by vehicles that can best satisfy the considered requirements. Nevertheless, there is no distinctly separate discussion of results since the explanation is rather focused toward the overall purpose of system.

### 4.1. Results of FS-AQoS

**Figure 8:**
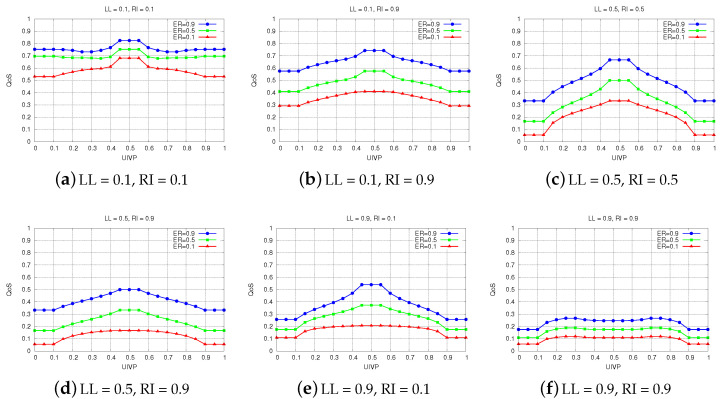
Simulation results for FS-AQoS.

The simulation results for FS-AQoS are presented in Figure 8. We see the effect of UIVP on QoS for different degrees of reliability, by considering many scenarios with various radio interference levels and link latency values.

Figure 8a gives the results for LL = 0.1 and RI = 0.1. We can see that in all cases, the QoS is decided above the moderate level. This is due to the fact that the communication links between vehicles have very low latency and there is no or very little interference present. However, when the interference is at harmful levels (see Figure 8b), the links that will be considered for a potential communication are mostly the ones with high reliability. The links with medium effective reliability, on the other hand, are considered only when vehicles are using a moderate number of UIVP packets. In all other cases, the QoS values are smaller than 0.5, which means that the chances of successful communications are not very high.

In Figure 8c, we consider the scenarios with LL = 0.5 and RI = 0.5. The results show clearly the importance of UIVP in QoS. A link with effective reliability is no longer enough to have successful communication, as opposed to the scenarios in Figure 8b, where links with high ER are considered for communication regardless of the number of UIVP packets. In these conditions, acceptable QoS values are achieved only for moderate levels of UIVP.

The same holds true for the scenarios in Figure 8d,e, with the difference that the links must have high reliability. If the link does not have high reliability, the QoS is decided under the moderate level, in any case. Comparing the results for these scenarios, we see that having no interference in reliable links with high latency (RI = 0.1, LL = 0.9) is slightly better than the other conditions (RI = 0.9 and LL = 0.5), as a vehicle being exposed under the former conditions can at least use the available communication links for applications that are not time-sensitive.

For both high latency and high interference levels (see Figure 8f), the QoS is always decided as extremely or very low in all cases. Under these circumstances, it is impossible for vehicles to use these communication links, as performing any kind of transmission between vehicles will lead to loss of information.

### 4.2. Results of FS-ANVPC

**Figure 9:**
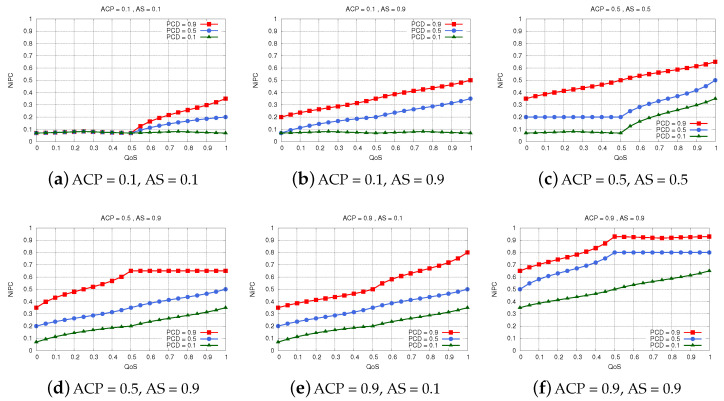
Simulation results for FS-ANVPC.

While we have conducted many simulations for FS-ANVPC, the scenarios we present in this paper are the ones shown in Figure 9. The results show the relation between QoS and NiPC for different PCD values. The effect of ACP or AS on NiPC can be seen when a pair of subfigures are selected for comparison, provided that one of the parameters has the same value.

In Figure 9a, we show the results for ACP = 0.1 and AS = 0.1. The results indicate that no neighbor vehicle can be of much help for *the vehicle* in these conditions. In Figure 9b, we see that there is some improvement in NiPC values when the neighbor vehicles have large amounts of storage resources to share. Nevertheless, the probability *the vehicle* uses the resources of these neighbors remains very low. This is can be attributed to the low ACP, which denotes that there will be no guarantee that these neighbors can run any additional applications successfully.

In Figure 9c,d, we see how an increase in ACP, however small, affects NiCP. A neighbor vehicle offering a medium amount of computing power has more chances of being selected to help *the vehicle*, provided that their connection is of high quality and they stay connected for a long time.

When the neighboring vehicles have much more to offer in terms of ACP, their NiPC values are improved even more. We can see this effect in Figure 9e,f. Nevertheless, if they have almost no storage to offer, the neighbors should be within the communication range of *the vehicle* for a long time and the QoS must be above the moderate level to enable quick data transmission all the time. On the other hand, when the neighbor vehicles are willing to and can share abundant resources in terms of both storage and computing power, we see that the system decides that they are capable of helping even if they do not stay connected with *the vehicle* for a long time. These neighbors are sometimes chosen even when the PCD is short or the QoS is not in high values, since letting their resources unexploited would lead to a huge amount of resources in the edge layer being unused.

### 4.3. Results of FS-CFELS

**Figure 10:**
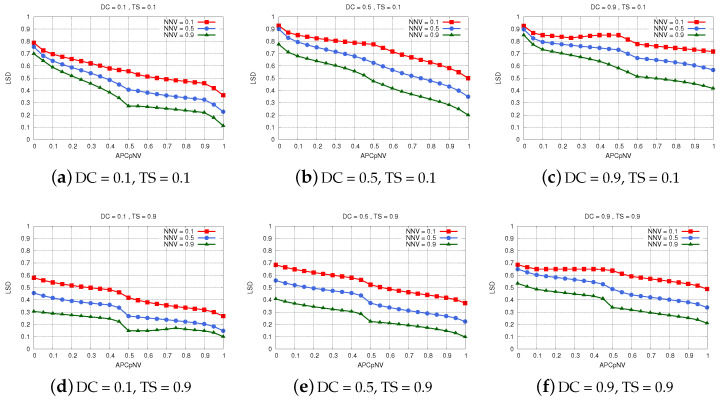
Simulation results for FS-CFELS.

The simulation results of FS-CFELS are given in Figure 10. The parameters considered constant for presenting the results are DC and TS as these parameters represent the application requirements, and as such, they differ only from application to application. Therefore, each subfigure represents practically a different set of applications that have similar requirements. Using this configuration we can see how LSD relates with the changing characteristics of the edge layer, which are represented by NNV and APCpNV.

The simulation results considering a set of non-complex applications that are delay tolerant are presented in Figure 10a. The results show that when *the vehicle* is surrounded by many potentially helpful neighbors, the system selects the edge layer as the most appropriate layer for *the vehicle* to run its applications. Although these applications do not require real-time processing, running them in the edge layer has two benefits: it exploits the high capacity of neighbors which at this point is being unused and it avoids unnecessary traffic being sent in the core network. On the other hand, in Figure 10b,c, we can see that the edge layer is hardly selected when the data complexity increases. Most of the data are sent in the fog or cloud layer, with the latter being used significantly more as the complexity increases. Using the cloud layer instead of fog for complex data frees the fog servers from unnecessary overload, considering the fact that these applications do not need to run in real-time.

However, as we can see from the results shown in Figure 10d–f, the system decides that the time-sensitive applications will be processed only in the edge and fog layer and never in the cloud. This decision fulfills such a strong requirement like latency. When the applications are not too complex, *the vehicle* is suggested to use mostly the resources of the neighboring vehicles, provided that there is a considerable number of them in its vicinity. When the number of neighboring vehicles is not very high or they are not prospective helpers, the system suggests the fog layer as the appropriate layer, especially for complex data. Fog servers have more powerful computing capabilities, and since they offer low latency as well, they can handle these data better while still ensuring real-time processing.

## 5. Conclusions

In this paper, we discussed the need for new strategies that can efficiently coordinate and manage the abundant resources available in VANETs and proposed an intelligent approach that can achieve this goal in a very flexible way. The proposed integrated fuzzy-based system decides the resources that vehicles should use when set under different circumstances. These circumstances include the condition of the network created among vehicles, which is represented by the QoS in the network, its longevity, its size, and the currently available resource, together with the application requirements, such as their complexity and time-sensitivity. We evaluated the proposed approach by computer simulations. From the simulation results, we conclude the following.

- Higher QoS values are achieved for a moderate number of beacon messages broadcasted, which increases the possibility of vehicles being categorized as potentially helpful neighbors.
- When a neighbor vehicle offers only a small amount of resources, it is considered less capable of helping, regardless of the quality of communication.
- In a dense environment, moderate complex data can be processed in the edge only if there are many potentially helpful neighbors in the vicinity.
- Time-sensitive applications are run either in edge or fog layer and never in the cloud.
- With the increase of data complexity less data is processed in the edge layer even if vehicles stay connected to the same potentially helpful neighbors for a long time.

In the future, we would like to improve IFS-CMR by considering parameters that characterize the fog layer and implementing the proposed approach in a testbed in order to demonstrate its accuracy.

## Figures and Tables

**Table 1:** Parameters and term sets for FS-AQoS.

Parameters	Term Sets
Link Latency (LL)	Low (Lo), Medium (Me), High (Hi)
Radio Interference (RI)	Permissible (Pe), Acceptable (Ac), Harmful (Ha)
Effective Reliability (ER)	Not Effective (Nef), Medium Effective (Mef), Effective (Ef)
Update Info. for Vehicle Position (UIVP)	Few (Fw), Moderate (Mo), Many (Ma)
Quality of Service (QoS)	Extremely Low (El), Very Low (Vl), Low (Lw), Moderate (Md),
	High (Hg), Very High (Vh), Extremely High (Eh)

**Table 2:** Parameters and term sets for FS-ANVPC.

Parameters	Term Sets
Available Computing Power (ACP)	Small (Sm), Medium (Me), Large (La)
Available Storage (AS)	Small (S), Medium (M), Big (B)
Predicted Contact Duration (PCD)	Short (Sh), Medium (Md), Long (Lo)
Quality of Service (QoS)	Low (Lw), Moderate (Mo), High (Hi)
Neighbor *i* Processing Capability (NiPC)	Extremely Low PC (ELPC), Very Low PC (VLPC),
	Low PC (LPC), Moderate PC (MPC), High PC (HPC),
	Very High PC (VHPC), Extremely High PC (EHPC)

**Table 3:** Parameters and term sets for FS-CFELS.

Parameters	Term Sets
Data Complexity (DC)	Low (Lo), Moderate (Mo), High (Hi)
Time Sensitivity (TS)	Low (Lw), Middle (Md), High (Hg)
Number of Neighboring Vehicles (NNV)	Sparse(Sp), Medium Density (Me), Dense (De)
Avg. PC per Neighbor Vehicle (APCpNV)	Low (L), Moderate (M), High (H)
Layer Selection Decision (LSD)	Decision Level 1 (DL1), DL2, DL3, DL4, DL5, DL6, DL7

**Table 4:** FRB of FS-AQoS.

No	LL	RI	ER	UIVP	QoS	No	LL	RI	ER	UIVP	QoS	No	LL	RI	ER	UIVP	QoS
1	Lo	Pe	Nef	Fw	Hg	28	Me	Pe	Nef	Fw	Vl	55	Hi	Pe	Nef	Fw	El
2	Lo	Pe	Nef	Mo	Eh	29	Me	Pe	Nef	Mo	Lw	56	Hi	Pe	Nef	Mo	Vl
3	Lo	Pe	Nef	Ma	Hg	30	Me	Pe	Nef	Ma	Vl	57	Hi	Pe	Nef	Ma	El
4	Lo	Pe	Mef	Fw	Vh	31	Me	Pe	Mef	Fw	Lw	58	Hi	Pe	Mef	Fw	El
5	Lo	Pe	Mef	Mo	Eh	32	Me	Pe	Mef	Mo	Md	59	Hi	Pe	Mef	Mo	Lw
6	Lo	Pe	Mef	Ma	Vh	33	Me	Pe	Mef	Ma	Lw	60	Hi	Pe	Mef	Ma	El
7	Lo	Pe	Ef	Fw	Eh	34	Me	Pe	Ef	Fw	Md	61	Hi	Pe	Ef	Fw	Vl
8	Lo	Pe	Ef	Mo	Eh	35	Me	Pe	Ef	Mo	Hg	62	Hi	Pe	Ef	Mo	Md
9	Lo	Pe	Ef	Ma	Eh	36	Me	Pe	Ef	Ma	Md	63	Hi	Pe	Ef	Ma	Vl
10	Lo	Ac	Nef	Fw	Md	37	Me	Ac	Nef	Fw	El	64	Hi	Ac	Nef	Fw	El
11	Lo	Ac	Nef	Mo	Hg	38	Me	Ac	Nef	Mo	Lw	65	Hi	Ac	Nef	Mo	El
12	Lo	Ac	Nef	Ma	Md	39	Me	Ac	Nef	Ma	El	66	Hi	Ac	Nef	Ma	El
13	Lo	Ac	Mef	Fw	Hg	40	Me	Ac	Mef	Fw	Vl	67	Hi	Ac	Mef	Fw	El
14	Lo	Ac	Mef	Mo	Eh	41	Me	Ac	Mef	Mo	Md	68	Hi	Ac	Mef	Mo	Vl
15	Lo	Ac	Mef	Ma	Hg	42	Me	Ac	Mef	Ma	Vl	69	Hi	Ac	Mef	Ma	El
16	Lo	Ac	Ef	Fw	Vh	43	Me	Ac	Ef	Fw	Lw	70	Hi	Ac	Ef	Fw	El
17	Lo	Ac	Ef	Mo	Eh	44	Me	Ac	Ef	Mo	Hg	71	Hi	Ac	Ef	Mo	Lw
18	Lo	Ac	Ef	Ma	Vh	45	Me	Ac	Ef	Ma	Lw	72	Hi	Ac	Ef	Ma	El
19	Lo	Ha	Nef	Fw	Lw	46	Me	Ha	Nef	Fw	El	73	Hi	Ha	Nef	Fw	El
20	Lo	Ha	Nef	Mo	Md	47	Me	Ha	Nef	Mo	Vl	74	Hi	Ha	Nef	Mo	El
21	Lo	Ha	Nef	Ma	Lw	48	Me	Ha	Nef	Ma	El	75	Hi	Ha	Nef	Ma	El
22	Lo	Ha	Mef	Fw	Md	49	Me	Ha	Mef	Fw	Vl	76	Hi	Ha	Mef	Fw	El
23	Lo	Ha	Mef	Mo	Hg	50	Me	Ha	Mef	Mo	Lw	77	Hi	Ha	Mef	Mo	El
24	Lo	Ha	Mef	Ma	Md	51	Me	Ha	Mef	Ma	Vl	78	Hi	Ha	Mef	Ma	El
25	Lo	Ha	Ef	Fw	Hg	52	Me	Ha	Ef	Fw	Lw	79	Hi	Ha	Ef	Fw	El
26	Lo	Ha	Ef	Mo	Vh	53	Me	Ha	Ef	Mo	Md	80	Hi	Ha	Ef	Mo	El
27	Lo	Ha	Ef	MaH	Hg	54	Me	Ha	Ef	Ma	Lw	81	Hi	Ha	Ef	Ma	El

**Table 5:** FRB of FS-ANVPC.

No	ACP	AS	PCD	QoS	NiPC	No	ACP	AS	PCD	QoS	NiPC	No	ACP	AS	PCD	QoS	NiPC
1	Sm	S	Sh	Lw	ELPC	28	Me	S	Sh	Lw	ELPC	55	La	S	Sh	Lw	ELPC
2	Sm	S	Sh	Mo	ELPC	29	Me	S	Sh	Mo	ELPC	56	La	S	Sh	Mo	VLPC
3	Sm	S	Sh	Hi	ELPC	30	Me	S	Sh	Hi	VLPC	57	La	S	Sh	Hi	LPC
4	Sm	S	Md	Lw	ELPC	31	Me	S	Md	Lw	VLPC	58	La	S	Md	Lw	VLPC
5	Sm	S	Md	Mo	ELPC	32	Me	S	Md	Mo	VLPC	59	La	S	Md	Mo	LPC
6	Sm	S	Md	Hi	VLPC	33	Me	S	Md	Hi	LPC	60	La	S	Md	Hi	MPC
7	Sm	S	Lo	Lw	ELPC	34	Me	S	Lo	Lw	VLPC	61	La	S	Lo	Lw	LPC
8	Sm	S	Lo	Mo	ELPC	35	Me	S	Lo	Mo	LPC	62	La	S	Lo	Mo	MPC
9	Sm	S	Lo	Hi	LPC	36	Me	S	Lo	Hi	MPC	63	La	S	Lo	Hi	VHPC
10	Sm	M	Sh	Lw	ELPC	37	Me	M	Sh	Lw	ELPC	64	La	M	Sh	Lw	VLPC
11	Sm	M	Sh	Mo	ELPC	38	Me	M	Sh	Mo	ELPC	65	La	M	Sh	Mo	LPC
12	Sm	M	Sh	Hi	ELPC	39	Me	M	Sh	Hi	LPC	66	La	M	Sh	Hi	HPC
13	Sm	M	Md	Lw	ELPC	40	Me	M	Md	Lw	VLPC	67	La	M	Md	Lw	LPC
14	Sm	M	Md	Mo	ELPC	41	Me	M	Md	Mo	VLPC	68	La	M	Md	Mo	MPC
15	Sm	M	Md	Hi	VLPC	42	Me	M	Md	Hi	MPC	69	La	M	Md	Hi	VHPC
16	Sm	M	Lo	Lw	ELPC	43	Me	M	Lo	Lw	LPC	70	La	M	Lo	Lw	MPC
17	Sm	M	Lo	Mo	VLPC	44	Me	M	Lo	Mo	MPC	71	La	M	Lo	Mo	VHPC
18	Sm	M	Lo	Hi	LPC	45	Me	M	Lo	Hi	HPC	72	La	M	Lo	Hi	EHPC
19	Sm	B	Sh	Lw	ELPC	46	Me	B	Sh	Lw	ELPC	73	La	B	Sh	Lw	LPC
20	Sm	B	Sh	Mo	ELPC	47	Me	B	Sh	Mo	VLPC	74	La	B	Sh	Mo	MPC
21	Sm	B	Sh	Hi	ELPC	48	Me	B	Sh	Hi	LPC	75	La	B	Sh	Hi	HPC
22	Sm	B	Md	Lw	ELPC	49	Me	B	Md	Lw	VLPC	76	La	B	Md	Lw	MPC
23	Sm	B	Md	Mo	VLPC	50	Me	B	Md	Mo	LPC	77	La	B	Md	Mo	VHPC
24	Sm	B	Md	Hi	LPC	51	Me	B	Md	Hi	MPC	78	La	B	Md	Hi	VHPC
25	Sm	B	Lo	Lw	VLPC	52	Me	B	Lo	Lw	LPC	79	La	B	Lo	Lw	HPC
26	Sm	B	Lo	Mo	LPC	53	Me	B	Lo	Mo	HPC	80	La	B	Lo	Mo	EHPC
27	Sm	B	Lo	Hi	MPC	54	Me	B	Lo	Hi	HPC	81	La	B	Lo	Hi	EHPC

**Table 6:** FRB of FS-CFELS.

No	DC	TS	NNV	APCpNV	LSD	No	DC	TS	NNV	APCpNV	LSD	No	DC	TS	NNV	APCpNV	LSD
1	Lo	Lw	Sp	L	DL6	28	Mo	Lw	Sp	L	DL7	55	Hi	Lw	Sp	L	DL7
2	Lo	Lw	Sp	M	DL4	29	Mo	Lw	Sp	M	DL6	56	Hi	Lw	Sp	M	DL7
3	Lo	Lw	Sp	H	DL3	30	Mo	Lw	Sp	H	DL4	57	Hi	Lw	Sp	H	DL6
4	Lo	Lw	Me	L	DL6	31	Mo	Lw	Me	L	DL7	58	Hi	Lw	Me	L	DL7
5	Lo	Lw	Me	M	DL3	32	Mo	Lw	Me	M	DL5	59	Hi	Lw	Me	M	DL6
6	Lo	Lw	Me	H	DL2	33	Mo	Lw	Me	H	DL3	60	Hi	Lw	Me	H	DL5
7	Lo	Lw	De	L	DL6	34	Mo	Lw	De	L	DL6	61	Hi	Lw	De	L	DL7
8	Lo	Lw	De	M	DL2	35	Mo	Lw	De	M	DL4	62	Hi	Lw	De	M	DL5
9	Lo	Lw	De	H	DL1	36	Mo	Lw	De	H	DL2	63	Hi	Lw	De	H	DL4
10	Lo	Md	Sp	L	DL5	37	Mo	Md	Sp	L	DL7	64	Hi	Md	Sp	L	DL7
11	Lo	Md	Sp	M	DL3	38	Mo	Md	Sp	M	DL5	65	Hi	Md	Sp	M	DL6
12	Lo	Md	Sp	H	DL2	39	Mo	Md	Sp	H	DL4	66	Hi	Md	Sp	H	DL5
13	Lo	Md	Me	L	DL4	40	Mo	Md	Me	L	DL6	67	Hi	Md	Me	L	DL7
14	Lo	Md	Me	M	DL2	41	Mo	Md	Me	M	DL4	68	Hi	Md	Me	M	DL5
15	Lo	Md	Me	H	DL1	42	Mo	Md	Me	H	DL3	69	Hi	Md	Me	H	DL4
16	Lo	Md	De	L	DL3	43	Mo	Md	De	L	DL5	70	Hi	Md	De	L	DL7
17	Lo	Md	De	M	DL1	44	Mo	Md	De	M	DL3	71	Hi	Md	De	M	DL4
18	Lo	Md	De	H	DL1	45	Mo	Md	De	H	DL2	72	Hi	Md	De	H	DL3
19	Lo	Hg	Sp	L	DL4	46	Mo	Hg	Sp	L	DL5	73	Hi	Hg	Sp	L	DL5
20	Lo	Hg	Sp	M	DL3	47	Mo	Hg	Sp	M	DL4	74	Hi	Hg	Sp	M	DL5
21	Lo	Hg	Sp	H	DL2	48	Mo	Hg	Sp	H	DL3	75	Hi	Hg	Sp	H	DL4
22	Lo	Hg	Me	L	DL3	49	Mo	Hg	Me	L	DL4	76	Hi	Hg	Me	L	DL5
23	Lo	Hg	Me	M	DL2	50	Mo	Hg	Me	M	DL3	77	Hi	Hg	Me	M	DL4
24	Lo	Hg	Me	H	DL1	51	Mo	Hg	Me	H	DL2	78	Hi	Hg	Me	H	DL3
25	Lo	Hg	De	L	DL2	52	Mo	Hg	De	L	DL3	79	Hi	Hg	De	L	DL4
26	Lo	Hg	De	M	DL1	53	Mo	Hg	De	M	DL2	80	Hi	Hg	De	M	DL3
27	Lo	Hg	De	H	DL1	54	Mo	Hg	De	H	DL1	81	Hi	Hg	De	H	DL2

## Data Availability

Not applicable.
